# ^18^F-Fluoride and ^18^F-Fluorodeoxyglucose Positron Emission Tomography After Transient Ischemic Attack or Minor Ischemic Stroke: Case–Control Study

**DOI:** 10.1161/CIRCIMAGING.116.004976

**Published:** 2017-03-14

**Authors:** Alex T. Vesey, William S. A. Jenkins, Agnese Irkle, Alastair Moss, Greg Sng, Rachael O. Forsythe, Tim Clark, Gemma Roberts, Alison Fletcher, Christophe Lucatelli, James H. F. Rudd, Anthony P. Davenport, Nicholas L. Mills, Rustam Al-Shahi Salman, Martin Dennis, William N. Whiteley, Edwin J. R. van Beek, Marc R. Dweck, David E. Newby

**Affiliations:** From the BHF Centre for Cardiovascular Science, University of Edinburgh, United Kingdom (A.T.V., W.S.A.J., A.M., G.S., R.O.F., N.L.M., E.J.R.v.B., M.R.D., D.E.N.); Division of Experimental Medicine and Immunotherapeutics, University of Cambridge, United Kingdom (A.I., J.R., A.P.D.); and Clinical Research Imaging Centre (T.C., G.R., A.F., C.L., E.J.R.v.B., M.R.D., D.E.N.) and Centre for Clinical Brain Sciences (R.A.-S.S., M.D., W.W.), University of Edinburgh, United Kingdom.

**Keywords:** carotid stenosis, fluorides, inflammation, nuclear medicine, phenotype, stroke

## Abstract

**Background—:**

Combined positron emission tomography (PET) and computed tomography (CT) can assess both anatomy and biology of carotid atherosclerosis. We sought to assess whether ^18^F-fluoride or ^18^F-fluorodeoxyglucose can identify culprit and high-risk carotid plaque.

**Methods and Results—:**

We performed ^18^F-fluoride and ^18^F-fluorodeoxyglucose PET/CT in 26 patients after recent transient ischemic attack or minor ischemic stroke: 18 patients with culprit carotid stenosis awaiting carotid endarterectomy and 8 controls without culprit carotid atheroma. We compared standardized uptake values in the clinically adjudicated culprit to the contralateral asymptomatic artery, and assessed the relationship between radiotracer uptake and plaque phenotype or predicted cardiovascular risk (ASSIGN score [Assessing Cardiovascular Risk Using SIGN Guidelines to Assign Preventive Treatment]). We also performed micro PET/CT and histological analysis of excised plaque. On histological and micro PET/CT analysis, ^18^F-fluoride selectively highlighted microcalcification. Carotid ^18^F-fluoride uptake was increased in clinically adjudicated culprit plaques compared with asymptomatic contralateral plaques (log_10_standardized uptake value_mean_ 0.29±0.10 versus 0.23±0.11, *P*=0.001) and compared with control patients (log_10_standardized uptake value_mean_ 0.29±0.10 versus 0.12±0.11, *P*=0.001). ^18^F-Fluoride uptake correlated with high-risk plaque features (remodeling index [*r*=0.53, *P*=0.003], plaque burden [*r*=0.51, *P*=0.004]), and predicted cardiovascular risk [*r*=0.65, *P*=0.002]). Carotid ^18^F-fluorodeoxyglucose uptake appeared to be increased in 7 of 16 culprit plaques, but no overall differences in uptake were observed in culprit versus contralateral plaques or control patients. However, ^18^F-fluorodeoxyglucose did correlate with predicted cardiovascular risk (*r*=0.53, *P*=0.019), but not with plaque phenotype.

**Conclusions—:**

^18^F-Fluoride PET/CT highlights culprit and phenotypically high-risk carotid plaque. This has the potential to improve risk stratification and selection of patients who may benefit from intervention.

## Abstract 2

Supplemental Digital Content is available in the text.

Although carotid endarterectomy reduces risk of ipsilateral stroke in people with symptomatic carotid artery stenosis, the number needed to treat to prevent one stroke is large,^1,2^ especially in asymptomatic stenosis.^3^ Furthermore, the pathological event that leads to cerebral thromboembolism (atherosclerotic plaque rupture) is not necessarily correlated with luminal stenosis severity.^4^ Other pathological features, such as inflammation, cell death, and microcalcification, are important in driving both plaque formation and instability.^5–7^ New imaging biomarkers of these processes are therefore needed to improve risk stratification and clinical decision-making. Such biomarkers could also assess the response of plaque biology to novel pharmacological interventions and provide a way of identifying culprit lesions in patients with multiple plaques.

**See Editorial by Tawakol et al**

**See Clinical Perspective**

Hybrid positron emission tomography and computed tomography (PET/CT) is a molecular imaging modality that has high sensitivity for noninvasive in vivo detection of radiolabeled biomolecules tuned to a variety of pathophysiological processes. In carotid atherosclerosis imaging, the most widely used tracer has been ^18^F-fluorodeoxyglucose (^18^F-FDG)^8–14^: Recently, we have described another radiotracer, ^18^F-fluoride, in atherosclerosis imaging.^15,16^ We^15–18^ and others^19–23^ have shown that this tracer has major potential in cardiovascular disease. ^18^F-Fluoride can highlight culprit plaque in patients after myocardial infarction and high-risk plaques in patients with apparently stable coronary heart disease.^16^ We have shown that this is because ^18^F-fluoride can highlight areas of microcalcification indicative of necrotic atheroma.^24^ The ability to identify high risk or culprit plaque in the cephalic circulation has the potential to improve risk stratification in patients at high risk of stroke with a view to more targeted interventions. Our study aims were to compare and contrast the identification of clinically adjudicated culprit and high-risk plaque at the carotid bifurcation using ^18^F-fluoride and ^18^F-FDG PET/CT.

## Methods

### Patient Population

Two cohorts of people with a recent transient ischemic attack (TIA) or minor ischemic stroke were recruited: a case cohort with a high-grade internal carotid artery stenosis (≥50% by North American Symptomatic Carotid Endarterectomy Trial^25^ criteria for men, ≥70% for women) scheduled to undergo carotid endarterectomy and a control cohort in whom the cause of stroke was not attributed to carotid atheroma. Participants were recruited from outpatient clinics in National Health Service Lothian between January 2013 and June 2014 (for exclusion criteria, see Appendix in the Data Supplement). Research ethics committee approval (National Health Service West of Scotland Research Ethics Committee: 12/WS/0227) and the written and informed consent of all participants were obtained.

### Baseline Assessment

Participants underwent clinical assessment at baseline including standard hematologic and biochemical indices. Serum C-reactive protein concentration was measured using the MULTIGENT CRP Vario assay on the high-throughput ARCHITECT system (Abbott Laboratories, Abbott Park, IL). Predicted cardiovascular risk was estimated using the ASSIGN score: a validated Scottish cardiovascular risk score that is similar to the Framingham risk score but includes additional factors, such as social deprivation and family history.^26^

### PET/CT Protocol

Static ^18^F-FDG PET/CT was acquired using a hybrid scanner (Biograph mCT, Siemens Medical Systems, Erlangen, Germany) 90 minutes after the intravenous administration of a target dose of 200 MBq. A rigid neck collar was fitted to minimize movement and standardize position. An attenuation-correction CT scan (nonenhanced, low dose 120 kV, 50 mAs) was then performed followed by PET acquisition covering 2 bed positions with the first upper bed centered over the carotid bifurcation in 3-dimensional mode for 20 minutes per bed. Patients were fasted for 6 hours before scanning.

^18^F-Fluoride PET/CT was undertaken the subsequent day 60 minutes after administering 250 MBq ^18^F-fluoride. A neck collar was fitted and an attenuation-correction CT scan was performed. This was followed by PET acquisition covering 2 similar bed positions to the ^18^F-FDG scan allowing 15 minutes per bed. A subset of 5 patients underwent fully dynamic ^18^F-fluoride PET/CT with pharmacokinetic analysis as described previously.^24^ Dynamic PET provides a quantitative assessment of uptake and these data were used to validate the semiquantitative static imaging data.

After PET acquisition, a CT carotid angiogram was performed without moving the subject (Care Dose 4D, 120 kV, 145 mA, rotation time 0.5 seconds, pitch 0.8. Contrast: 50 mL Niopam 370).

Static PET data were reconstructed using the Siemens UltraHD algorithm: ordered subset expectation maximization+point spread function modeling+time-of-flight; 2 iterations and 21 subsets; matrix size 200×200; 5 mm full-width half-maximum Gaussian smoothing. Dynamic PET data were similarly reconstructed but only using coincident events from the 60- to 75-minute time-bin. Dynamic data were analyzed as reported previously^24^ and a *K*_*i*_ value was calculated using Patlak analysis.^27,28^

### Tissue Collection, Micro PET/CT, and Histology

At the time of endarterectomy, plaques were collected immediately after excision, photographed, and snap frozen. A random selection (n=8) of specimens was analyzed by micro PET/CT and histology to explore ^18^F-fluoride binding patterns (see Appendix in the Data Supplement for detailed methods).

## Image Analysis

### Positron Emission Tomography/Computed Tomography

Static analysis of ^18^F-FDG and ^18^F-fluoride uptake was performed on an OsiriX workstation (OsiriX version 3.5.1 64-bit; OsiriX Imaging Software, Geneva, Switzerland). PET/CT data were reviewed alongside the CT angiogram. Scans were qualitatively assessed for registration, image quality, patient movement, and visual evidence of radiotracer uptake. PET and CT data were individually and carefully manually coregistered by lining up fiducial markers apparent on both modalities (eg, cervical spine, mandible and hyoid on ^18^F-fluoride imaging; skin, spinal cord, and brain on ^18^F-FDG imaging). No formal inter-PET registration was performed. Three regions of interests (ROIs) were drawn on the carotid of interest on adjacent 3-mm axial slices. If a plaque was present, the ROIs were centered on the area of highest uptake. If there was no plaque, the uptake in the proximal 1 cm of internal carotid artery, just distal to the bifurcation was quantified. From these, standardized uptake values (SUVs; maximum, mean maximum, and mean) were recorded. Blood pool activity was determined from the average of 5 ROIs within the lumen of the superior vena cava to calculate target to background ratios.

Uptake in the proximal left common carotid artery was quantified to explore the relationships between arterial ^18^F-FDG and ^18^F-fluoride uptake in a site unaffected by an acute plaque event. Three ROIs were placed around this vessel and uptake was recorded.

Inter- and intraobserver reproducibility of ^18^F-fluoride uptake measurements were determined using a random selection of 12 patients (24 carotids) by 2 experienced observers (A.T.V., G.S.) who were blinded to the clinical data during analysis.

### Computed Tomography

The CT angiogram was assessed for image quality, plaque presence, location, and characteristics. Analysis was undertaken on a cardiovascular workstation (Vital Images, Minnetonka, MN). A blinded and experienced observer (A.V.) performed the semiautomated CT plaque analysis.

### Statistical Analysis

Radiotracer uptake, expressed as mean and maximum SUV, was compared between the clinically adjudicated culprit carotid plaque and the contralateral side. Continuous variables are expressed as mean±standard deviation for normally distributed data and median (interquartile range) for skewed distributions. Skewed datasets underwent logarithmic transformation to normalize their distribution. Parametric (unpaired and paired *t*-tests) and nonparametric (Mann–Whitney *U* or Wilcoxon matched-pairs signed rank) tests were used for normally distributed and skewed data, respectively. Categorical data are presented as n (%) and were compared using Fisher’s exact or Chi-squared tests. Correlation was undertaken with either Pearson’s *r* or Spearman’s *ρ* subject to the normality of the variables tested. To quantify inter- and intraobserver reproducibility of ^18^F-fluoride uptake measurement, the intraclass correlation coefficient was calculated and Bland-Altman analysis was undertaken.

Statistical analyses were performed with the use of SPSS version 18 (SPSS Inc, Chicago, IL) and Graph Pad Prism version 6.0 (GraphPad Software Inc, San Diego, CA). Statistical significance was defined as a 2-sided *P*<0.05.

## Results

### Study Population

**Figure 1.**
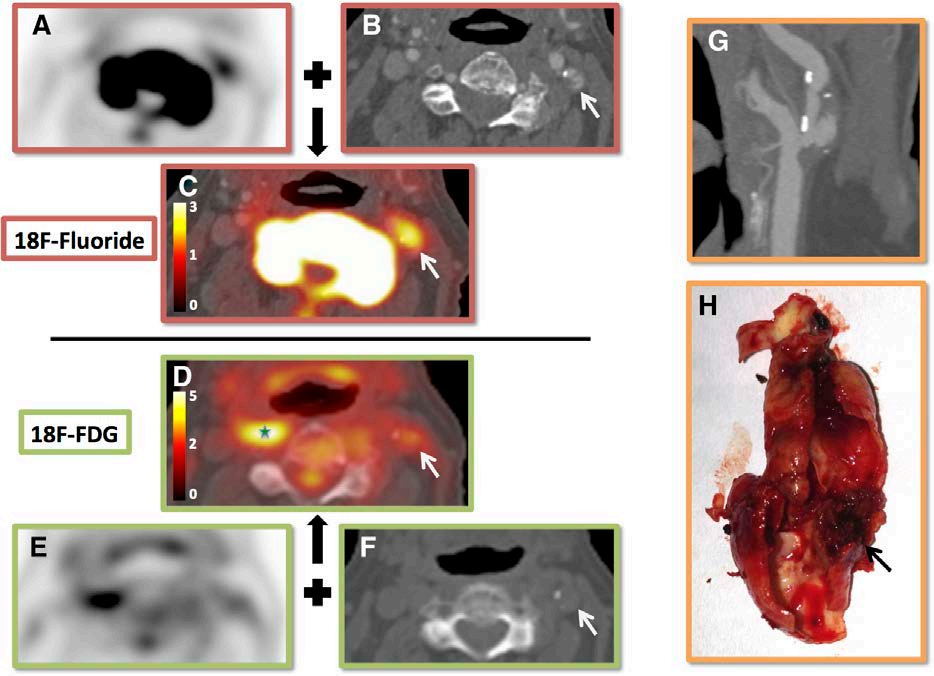
^18^F-Fluoride and ^18^F-fluorodeoxyglucose (FDG) positron emission tomography of carotid arteries. Example of ^18^F-fluoride (**A**, **B**, **C**) and ^18^F-FDG (**D**, **E**, **F**) positron emission tomography (PET)/computed tomography (CT) of 1 patient before surgery for symptomatic carotid stenosis. **A**, ^18^F-Fluoride PET axial slice. **B**, Registered CT angiogram axial slice. **C**, Fused PET/CT image. White arrow, Ruptured plaque showing ^18^F-fluoride uptake. **D**–**F**, Same slice but with ^18^F-FDG. Culprit shows uptake, but the contralateral side is obscured by uptake in the right longus colli (green star). An oblique computed tomography carotid angiogram reformat of the culprit (**G**). The operative specimen (**H**).

We recruited 26 patients: 18 in the carotid endarterectomy cohort and 8 in the control cohort (Figure I in the Data Supplement). Baseline characteristics (Table 1) were similar in both cohorts. Twenty patients completed all the imaging techniques (Figure 1). A minority did not receive all scans because of the technical and feasibility challenges of completing our multimodality imaging protocol in the very short time frame before surgery. Actual doses and uptake times are specified in Table I in the Data Supplement. There were no adverse events during the study. There were 3 withdrawals.

**Table 1.**
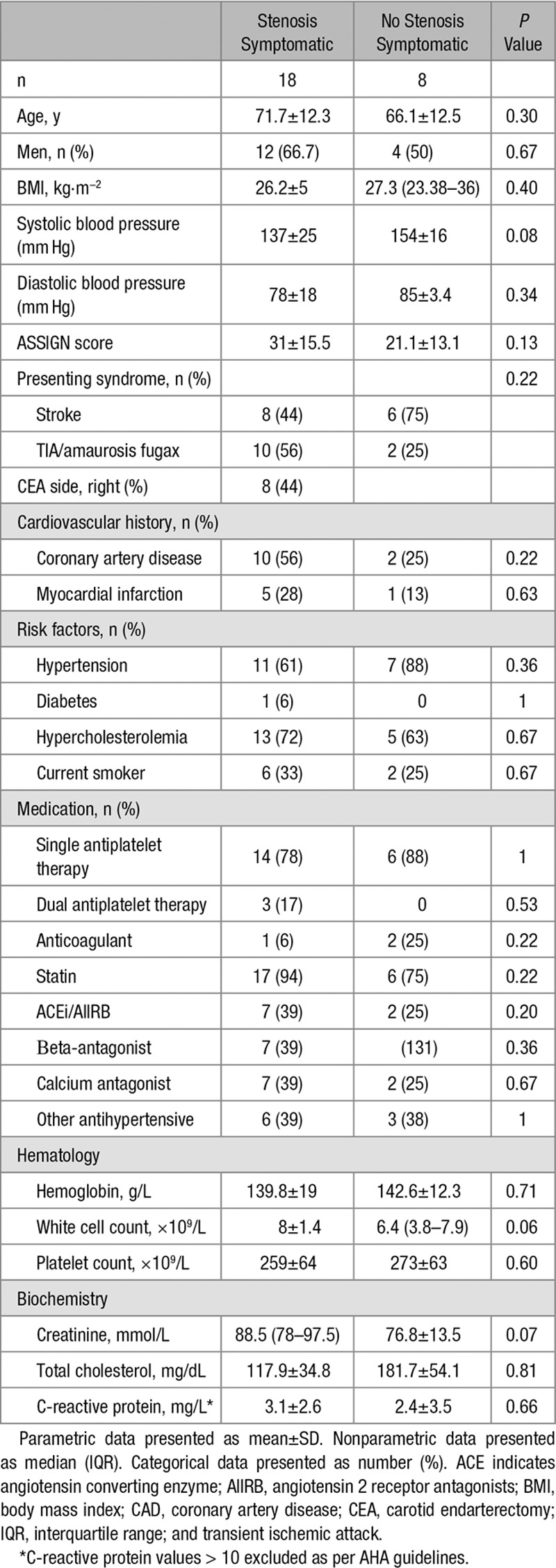
Baseline Clinical Characteristics

### Micro PET/CT and Histology

**Figure 2.**
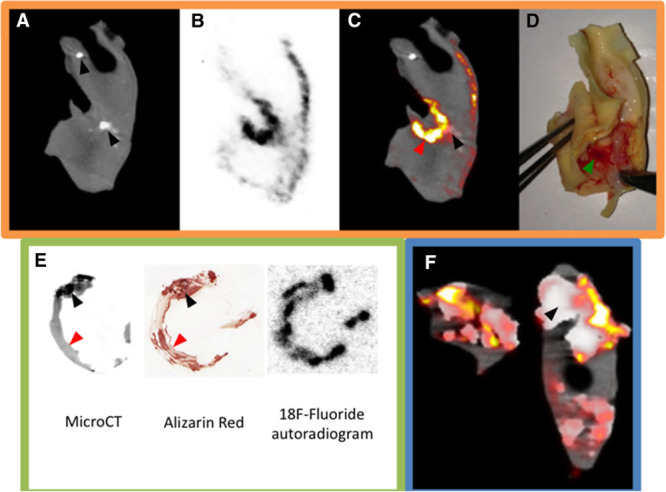
^18^F-Fluoride micro positron emission tomography (PET)/computed tomography (CT), autoradiography, and alizarin red staining. Two examples of ex vivo ^18^F-fluoride micro PET/CT are shown (**A**–**D**, **F**). **A**, Coronal micro CT slice; **B**, corresponding micro PET; **C**, fused image; **D**, the plaque. Green arrow, Adherent thrombus over plaque rupture. Red arrow, Associated area of ^18^F-fluoride uptake (microcalcification). Black arrows, Areas of macrocalcification showing comparatively little uptake (**A**, **C**, **F**). These examples show that ^18^F-fluoride provides information of the presence of microcalcification and does not simply highlight all calcification. **E**, An example of micro CT slice registered to an alizarin red-stained section and the corresponding autoradiogram from a specimen that had been incubated whole in ^18^F-fluoride. It can be seen that the tracer is unable to penetrate the deeper layers of macrocalcification (black arrow), but is able to highlight microcalcification beyond the resolution of even micro CT (red arrow), thus explaining the findings in the micro PET/CT images.

^18^F-Fluoride was observed to selectively highlight areas of pathologically high-risk microcalcification (Figure 2 and Supplementary Movie I in the Data Supplement). Both on autoradiography and micro PET/CT, ^18^F-fluoride was observed to bind avidly to areas of microcalcification but only to the surface of large volume stable macrocalcifications. Our previous studies^24^ would suggest that this was because of the inability of the fluoride ion to penetrate to the deeper layers of a large crystalline mass (with a low surface-area-to-volume ratio). In contradistinction, the powdery deposits of microcalcification (not visible on CT) provide a large area (high surface-area-to-volume ratio) for the fluoride ion to bind.

### Imaging

**Figure 3.**
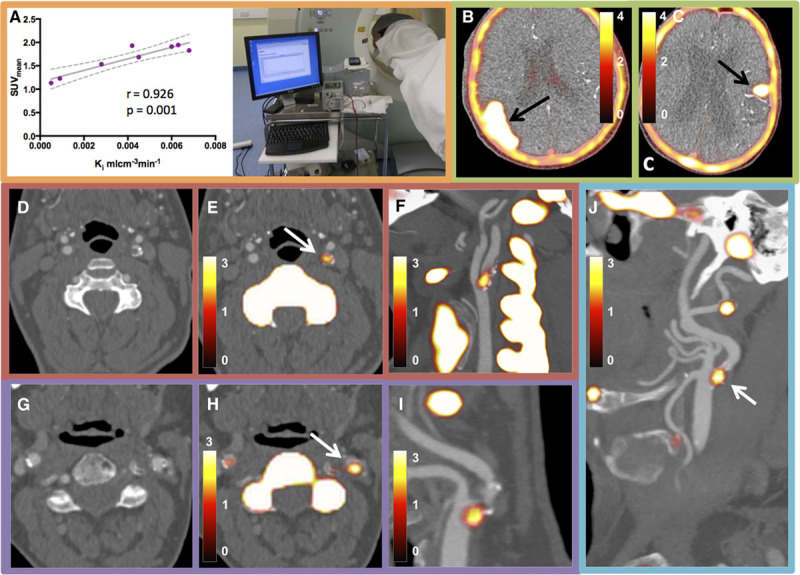
Dynamic positron emission tomography (PET) acquisition and examples of ^18^F-fluoride uptake. **A**, Correlation between statically derived standardized uptake value (SUV)_mean_ and dynamically measured *K*_*i*_ (dotted line is 95% confidence interval). Photograph shows a dynamic PET study in process. **B**, **C**, ^18^F-Fluoride uptake into areas of cerebral infarction. **D**–**F**, From 1 patient. **D**, Axial image from computed tomography carotid angiogram; **E**, Fused axial ^18^F-fluoride PET/computed tomography (CT; white arrow, culprit plaque); **F**, Oblique reconstruction. **G**–**I**, Similar reconstructions from a different patient. **J**, Obliquely reformatted PET/CT image from a patient who developed a fatal stroke (ipsilateral to the lesion marked by a white arrow) 2 weeks after this scan. The contralateral side, which had shown minimal uptake, had been deemed the culprit based on duplex assessment.

When comparing the ^18^F-fluoride uptake on static imaging with full dynamic modeling, *K*_*i*_ was most strongly correlated with the SUV_mean_ (*r*=0.93 [95% confidence interval 0.64–0.99], *P*=0.001; Figure 3). There were no fixed or proportional biases in the SUV measurements within and between observers (Table II in the Data Supplement). These assessments also demonstrated high intraclass correlation coefficients (all >0.90).

### Assessment of Uptake: Culprit Compared With Contralateral and Controls

**Figure 4.**
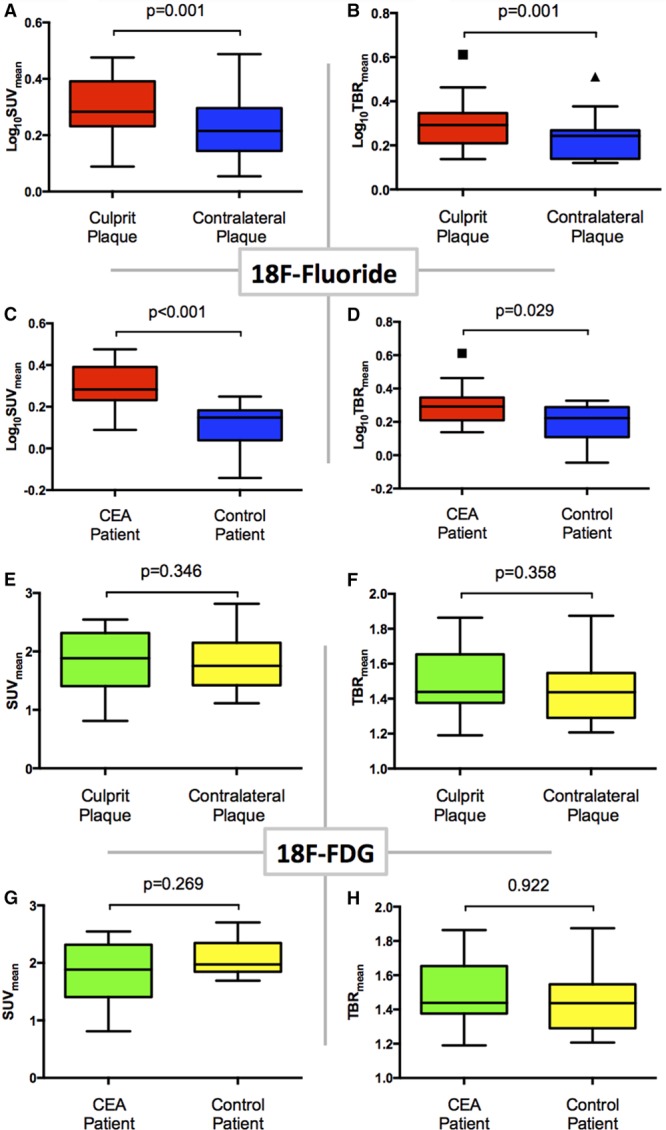
^18^F-Fluoride and ^18^F-fluorodeoxyglucose (FDG) positron emission tomography (PET)/computed tomography uptake. Dynamic PET acquisition and examples of ^18^F-fluoride uptake. Uptake in clinically adjudicated culprit vs contralateral and vs controls. Tukey box and whisker plots. **A**, **B**, ^18^F-Fluoride uptake into culprit (red) and contralateral (blue) plaque using the standardized uptake value (SUV)_mean_ and target to background ratio (TBR)_mean_ measurements, respectively. **C**, **D**, Each demonstrate comparison in ^18^F-fluoride uptake between carotid endarterectomy (CEA) patients (red) and controls (blue); uptake is reported by SUV_mean_ in **C** and TBR_mean_ in **D**. **E**–**H**, The same comparisons but using ^18^F-FDG.

^18^F-Fluoride uptake was variably present in most plaques with all culprits showing uptake on visual assessment. In the large majority of patients undergoing carotid endarterectomy who were scanned (87%; 13/15), there was more visual uptake of ^18^F-fluoride in the culprit compared with the contralateral side. In the 2 patients without discriminatory uptake, there was heavy uptake bilaterally but more ^18^F-fluoride uptake on the contralateral side. One patient had grossly ossified carotids and the second, at the time of surgery, was found to have a fibrous stenosis (low signal side) and was subsequently admitted with a fatal ischemic stroke on the contralateral side (high signal side, Figure 3J). ^18^F-Fluoride uptake was focal and readily identifiable with excellent signal to background discrimination. Spillover from the hyoid bone, thyroid cartilage and cervical vertebrae occasionally made drawing ROI difficult, but only 1 vessel was rendered uninterpretable. On SUV analysis, the clinically adjudicated culprit showed higher uptake than either the paired contralateral (log_10_SUV_mean_ 0.29±0.10 versus 0.23±0.11, *P*=0.001) or an unpaired control (log_10_SUV_mean_ 0.29±0.10 versus 0.12±0.11, *P*=0.001) irrespective of the method of quantification (Table 2 and Figures 3 and 4).

**Table 2.**
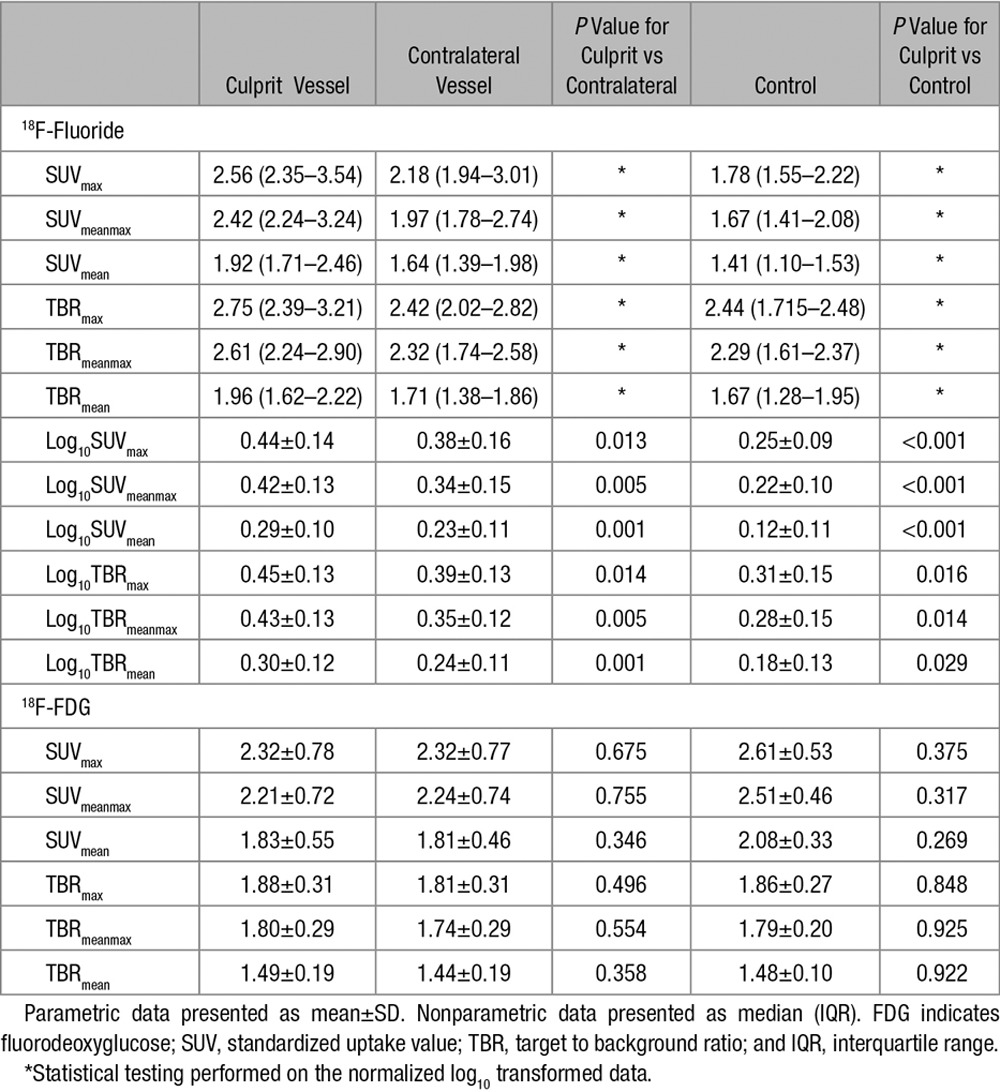
Radiotracer Uptake: Comparative Data

Of note, in patients with a stroke in whom the imaging extended to encompass the affected territory of the brain (n=3), intense ^18^F-fluoride uptake was noted in regions of cerebral infarction (SUV_mean_ 4.8±1.98 versus SUV_mean_ of 0.07±0.02 for contralateral noninfarcted brain, *P*<0.001; Figure 3B and 3C, Movie II in the Data Supplement).

Seven of the 16 culprit carotid plaques demonstrated clear and discernible increased ^18^F-FDG uptake. However, this uptake was generally more diffuse than ^18^F-fluoride and analysis was more frequently hampered by overspill from sternocleidomastoid, longus colli, tonsillar tissue, and the submandibular salivary glands (Figure 1). This rendered 5 vessels noninterpretable. In the remaining 4 culprit vessels, no increase in ^18^F-FDG uptake could be observed. Overall on semiquantitative analysis, ^18^F-FDG uptake was not higher in the clinically adjudicated culprit compared with either the paired contralateral (SUV_mean_ 1.83±0.55 versus 1.81±0.46, *P*=0.269) or control vessels (SUV_mean_ 1.83±0.55 versus 2.08±0.33, *P*=0.269) irrespective of the method of quantification (Table 2 and Figure 4).

### Uptake Compared With Plaque Features and Baseline Characteristics

^18^F-Fluoride uptake was correlated with several plaque characteristics on CT plaque analysis (Table 3). The strongest correlation was with the Agatston score (SUV_mean_ *r*=0.72, *P*<0.001), although there were also strong correlations with high-risk features such as plaque burden (SUV_mean_ *r*=0.51, *P*=0.003) and positive remodeling (wall-distal internal carotid artery lumen ratio, with SUV_mean_ *r*=0.53, *P*=0.003).

**Table 3.**
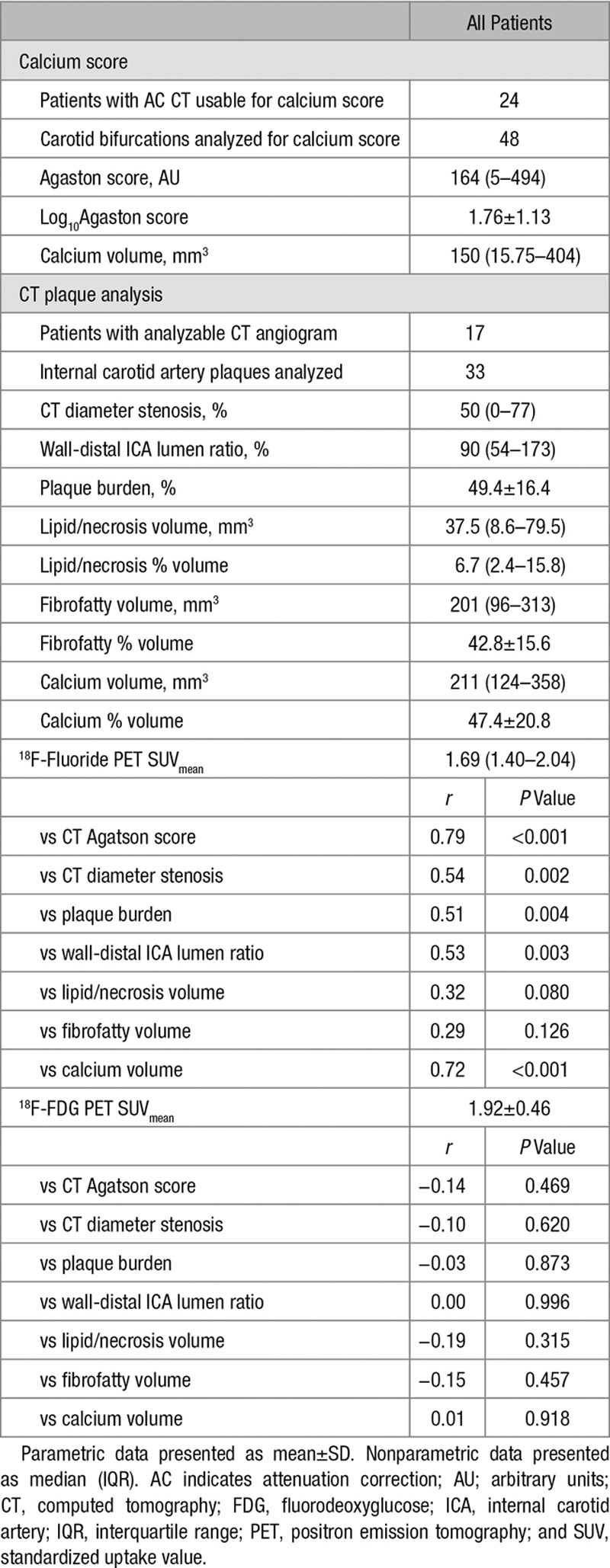
Plaque Analysis by CT and PET

In terms of baseline cardiovascular risk indices, uptake of both tracers in the vasculature correlated with age (^18^F-FDG SUV_meanmax_ *r*=0.48, *P*=0.037; ^18^F-fluoride SUV_mean_ *r*=0.59, *P*=0.007) and the cardiovascular risk score (^18^F-FDG SUV_meanmax_ *r*=0.53, *P*=0.019; ^18^F-fluoride SUV_mean_ *r*=0.65, *P*=0.002) but neither was associated with serum C-reactive protein concentration.

## Discussion

We have shown that the culprit plaques of patients with recent TIA or minor ischemic strokes enhance with ^18^F-fluoride on PET/CT. Uptake was focal, readily identifiable, and discriminated between culprit and nonculprit. ^18^F-Fluoride uptake was associated with high-risk plaque phenotype and predicted cardiovascular risk. In contrast, while ^18^F-FDG uptake was present in plaque and correlated with cardiovascular risk, it was more diffuse and prone to spillover and therefore less discriminatory. ^18^F-FDG also failed to correlate with established high-risk plaque morphological features.

We have previously shown that ^18^F-fluoride uptake is associated with increased intraplaque markers of cell death, procalcific proteins, inflammation, and high-risk features in the coronary circulation in vivo and the carotid system ex vivo.^16^ Here, we confirm our previous observations^24^ (which we have also recently reviewed^29^) that this is explained by the ability of ^18^F-fluoride to report microcalcification. Why is this the case? Far from a passive and degenerative process, vessel mineralization is a controlled response to a variety of insults, particularly oxidized inflammatory lipid (as in the calcific response to tuberculosis infection where lipid-rich bacterial cell walls become oxidized through leukocyte action). It is therefore perhaps no surprise that direct links between atherosclerosis and the induction of extraskeletal osteogenesis have been identified.^30,31^ The presence of cellular necrosis and apoptosis^32^ is also likely to potentiate this relationship further. Hydroxyapatite nanocrystals themselves may also further drive the inflammatory cycle by setting up a positive feedback loop of increasing calcification, increasing inflammation, and increasing cell death.^30^ Furthermore, by accumulating in the surface of thin fibrous caps, microcalcifications may focally increase mechanical stress and thus promote structural cap failure and plaque rupture.^7,33,34^ ^18^F-Fluoride can demonstrate this pathologically important microscopic calcific response.

This is the first observation of ^18^F-fluoride uptake in necrotic brain tissue and merits consideration. Uptake of this and other bone metabolism markers has previously been observed in tissue necrosis.^33,35^ This is likely to be because of cell membrane disruption with influx of calcium and formation of nanoscale calcium phosphate complexes. These offer a substrate to which ^18^F-fluoride can adsorb, allowing us to visualize the microcalcification associated with necrosis. We have also observed the same process in myocardial tissue postinfarction (Figure II in the Data Supplement).

We confirmed identification of culprit plaque in 2 ways. First, we compared the culprit to the ideal internal control, the contralateral carotid artery (which is almost invariably diseased). Second, we compared the culprit against a valid external control; patients with a TIA or minor ischemic stroke not attributed to carotid plaque. This shows that ^18^F-fluoride may have real potential in helping to identify culprit plaque thus helping decision-making. This is exemplified by the case where a plaque with high uptake deemed nonculprit subsequently caused a fatal ischemic stroke.

We compared uptake of ^18^F-fluoride with ^18^F-FDG. Unlike ^18^F-fluoride, overall, ^18^F-FDG uptake was not significantly higher in culprit lesions. Moreover, on a per-lesion basis, ^18^F-FDG failed to correlate with high-risk plaque morphological features, whereas ^18^F-fluoride uptake correlated with plaque burden, positive remodeling, and luminal stenosis: all established markers of plaque risk. Other studies have explored the utility of ^18^F-FDG alone in carotid atherosclerosis^9–11,14,36–39^ and a few have directly compared clinical culprit with nonculprit plaques.^8,12,13^ Our results are consistent with these previous findings with significant uptake noted in some but not all culprit plaques, in part because of spillover from adjacent muscle. Our observations are also influenced by the ubiquity of statin therapy, potentially blunting ^18^F-FDG uptake. We did, however, note that proximal carotid uptake correlated with cardiovascular risk indicating that ^18^F-FDG does reflect a major aspect of vessel pathobiology. As others suggest,^38,40^ it may be that ^18^F-FDG better reflects generalized vascular inflammation and that the relationship between the tracer and a single advanced and acute plaque is more complicated. There are increasing data available concerning other more specific markers of inflammation, such as those targeting the macrophage-specific somatostatin receptor.^41^ These will theoretically be less hampered by overspill.

Our findings confirm those of a smaller study of 9 patients by Quirce et al^23^ that explored ^18^F-fluoride and ^18^F-FDG uptake in symptomatic patients. They showed that ^18^F-fluoride uptake appeared to be higher in the symptomatic carotid and that 18-FDG uptake was nondiscriminatory. Taken together with our current larger series, this suggests that ^18^F-fluoride has the potential to be a useful and robust clinical tool to identify culprit atherosclerotic plaque. Vascular ^18^F-fluoride imaging could therefore guide clinical management better than the current standard of care, and lead to trials of plaque-specific interventions that go beyond simple assessments of anatomic luminal stenosis severity.

### Limitations

This was a small pilot observational study (recruitment is very challenging given the time pressure to intervene) and findings should be regarded as preliminary. The true utility of ^18^F-fluoride PET/CT will need to be evaluated by prospective studies with patients randomized to intervention based on imaging. ^18^F-Fluoride PET/CT will need to be compared with other techniques^42^ (in particular MR or PET/MR) which have the advantages of improved soft tissue definition, reduced radiation, and lack of iodinated contrast. We did not perform prolonged-delayed ^18^F-FDG imaging which some authors have suggested is advantageous.^43^ We also acknowledge that quantitative vascular PET has some potential limitations because of the partial volume effects of small vascular structures. Finally, as vascular ^18^F-fluoride imaging is developed, consideration must be given to harmonizing acquisition and reconstruction protocols,^44^ as well as achieving consensus on the uptake parameter of choice (SUV versus target to background ratio versus volumetric parameters^45^) and whether to use manual or automated methods to define ROI. This will reduce variation between scanners and research groups and permit meaningful multicenter studies.

## Conclusion

We have shown that ^18^F-fluoride PET/CT is able to identify culprit or high-risk carotid plaque. In comparison, ^18^F-FDG, the most widely used tracer in cardiovascular PET imaging, did not reliably identify culprit plaque and did not correlate with high-risk morphological features. ^18^F-Fluoride PET has major potential to improve how we assess and manage the risk of stroke in patients with atherosclerosis.

## Acknowledgments

We acknowledge the help and support of the vascular surgical staff at the Royal Infirmary of Edinburgh and the radiography and radiochemistry staff of the Clinical Research Imaging Centre.

## Sources of Funding

Dr Vesey and the study were funded by program grants from the British Heart Foundation (PG12/8/29371) and Chest Heart and Stroke Scotland (R13/A147). Dr Jenkins, Vesey, Dweck, and Newby are supported by the British Heart Foundation (FS/14/78/31020, CH/09/002) and the Wellcome Trust (WT103782AIA). Dr Dweck is the recipient of the Sir Jules Thorn Biomedical Research Award 2015. The Wellcome Trust Clinical Research Facility and the Clinical Research Imaging Centre are supported by National Health Service (NHS) Research Scotland (NRS) through NHS Lothian. Dr Beek is supported by the Scottish Imaging Network—a Platform of Scientific Excellence (SINAPSE). Dr Rudd is part-supported by the National Institute for Health Research Cambridge Biomedical Research Centre, the British Heart Foundation, and the Wellcome Trust.

## Disclosures

None.

## Supplementary Material

## CLINICAL PERSPECTIVE

Stroke remains the leading global cause of disability and is responsible for huge healthcare costs. It is commonly caused by thromboembolism from extracranial atherosclerotic plaque. In addition to medical therapy, invasive carotid artery intervention (by endarterectomy or stenting) has a role in reducing the chances of subsequent stroke. However, intervention itself is associated with significant risk and the decision to proceed with surgery is still based principally on stenosis severity, an outdated parameter. This report shows that by selectively demonstrating intraplaque microcalcification, a pathologically high-risk process that reflects ongoing inflammation and cell death, ^18^F-fluoride PET/CT may be able to identify plaque at particular risk of causing future stroke. The technique, as part of a multimodal risk stratification strategy, may help to ensure the appropriate planning of surgical intervention. This would potentially avoid unnecessary surgery on quiescent yet tightly stenosed plaques and conversely permit the identification and removal of high-risk plaques that currently do not meet criteria for intervention. ^18^F-Fluoride PET/CT also offers the possibility of noninvasively assessing the response to existing or novel pharmacological agents permitting the personalization of therapy to maximize benefit and minimize risk of complications.
