# Supplementary material for: 18F-Fluoride and 18F-Fluorodeoxyglucose Positron Emission Tomography After Transient Ischemic Attack or Minor Ischemic Stroke: Case–Control Study
Source: Circ Cardiovasc Imaging. 2017 Mar 14;10(3):e004976. doi: 10.1161/CIRCIMAGING.116.004976 (PMC5367506; doi:10.1161/CIRCIMAGING.116.004976)
Supplement: Supplementary file 1 [file hci-10-e004976-s001.pdf]

Supplemental Material

Supplemental Methods

Exclusion criteria

A modified Rankin score >3 (due to the burden of participation in those with limited mobility), insulin-dependent diabetes mellitus (due to the variability of 18F-FDG uptake), women of child-bearing potential, severe chronic kidney disease (estimated glomerular filtration rate <30 mL/min/1.73 m^2), known allergy to iodine-based contrast media, prior ipsilateral internal carotid artery intervention, prior neck irradiation, or those unable to provide informed consent.

MicroPET/CT

Carotid artery specimens were stored at -80 degree Celsius following removal during carotid endarterectomy. Thawed non-decalcified carotid artery specimens were incubated for 60 minutes in 18F-sodium fluoride 104.89kBq/mL solution (10.5MBq 18F-NaF in 99.5mLs 0.9% NaCl). Samples were twice washed in 100mLs 0.9% NaCl for 2 minutes to remove unbound 18F-Fluoride. Carotid artery specimens were scanned using high-resolution micro-positron emission tomography and non-contrast computed tomography [50kVp tube voltage, 300msec exposure time] (Mediso nanoScan PET/CT, Mediso Medical Imaging Systems, Hungary). PET-CT images were analysed on an OsiriX workstation (OsiriX version 7.5.1, 64-bit, OsiriX Imaging Software, Geneva, Switzerland).

Autoradiography and Histology

To perform 18F-fluoride autoradiography, 18F-fluoride was diluted to 1 x 10-11 M. Whole carotid plaque specimens were thawed in 5 mL PBS for 1 h, and then placed in 5 mL of the diluted 18F-fluoride solution for 1 h at room temperature. They were then washed in PBS three time and dipped in distilled water. Specimens was then embedded in the OCT Compound (CellPath, Powys, UK) and 20 µm thick, serial sections were cut on a Bright (Huntingdon, UK) cryostat and placed on Superfrost Plus slides (VWR, Lutterworth, UK). After drying, sections were placed on a charged phosphor screen (Perkin Elmer, Waltham, Massachusetts) and left overnight. The next day screens were read using PerkinElmer's Cyclone Plus Phosphor Imager (Waltham, Massachusetts) and data analysed with OptiQuantTM software (Packard Instrument, Meriden, Connecticut).

Alizarin Red (Alfa Aesar, Heysham, UK) was employed to stain calcium. Sections were fixed in acetone (4 °C) for ten minutes then washed in PBS at room temperature. After washing, 300 µL Alizarin Red was applied to each section for one minute. Samples were then transferred to acetone for 1 min, before being washed, in acetone:xylene (50:50) for 1 min. Sections were then incubated in xylene for at least 1 h. Tissue was then mounted using DePeX mounting medium Gurr (VWR, Lutterworth, UK) and glass coverslips (Menzel-Gläser, Braunschweig, Germany) were applied. Slides were imaged using Wild Heerbrugg M3Z microscope (Leica, Heerbrugg, Switzerland).

Supplemental Figures

Supplementary Figure 1: Study Flow Chart

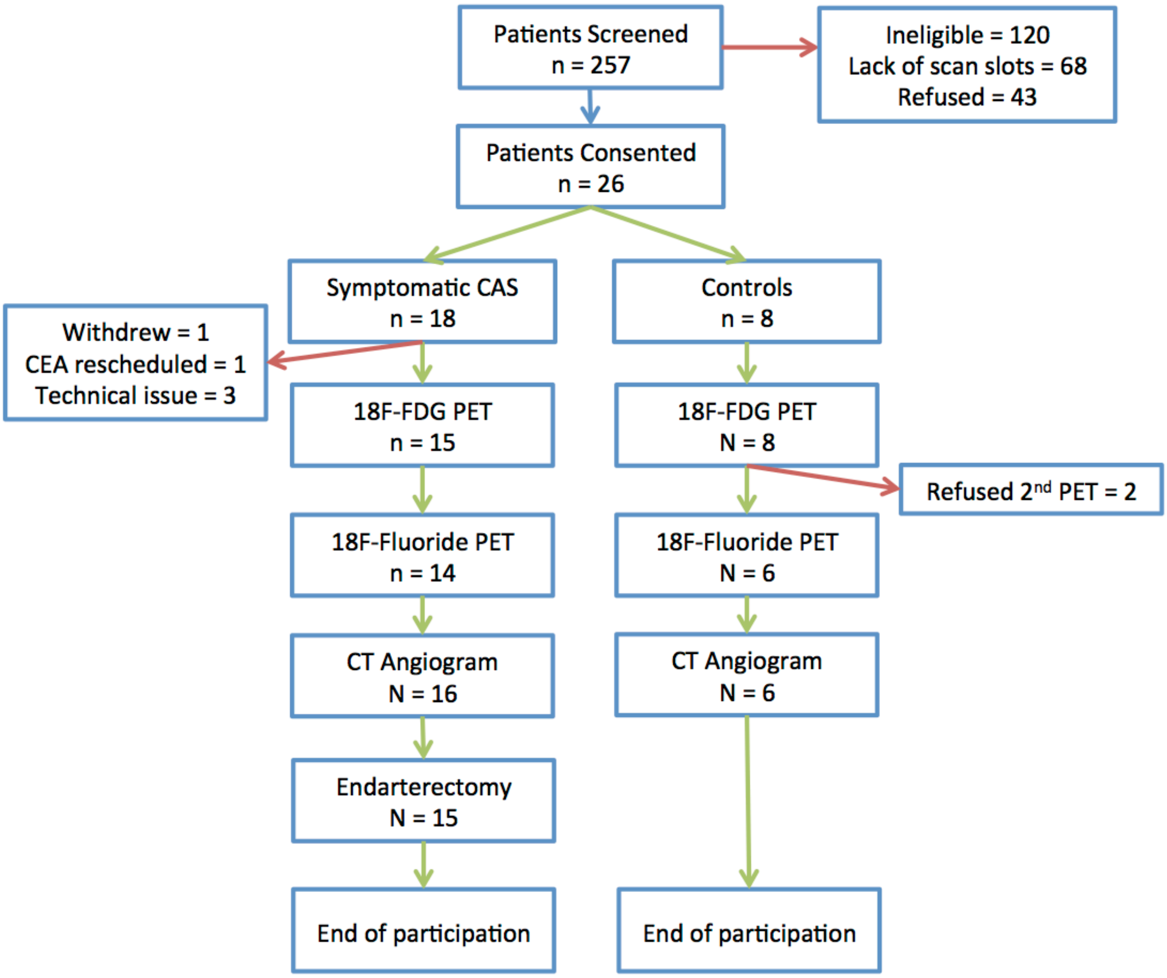

Abbreviations: CAS, carotid artery stenosis, CEA, carotid endarterectomy; CT, computed tomography; FDG, fluorodeoxylucose; PET, positron emission tomography.

Supplementary Figure 2: 18F-Fluoride Uptake in a Patient with a Large Anterior ST-elevation Myocardial Infarction

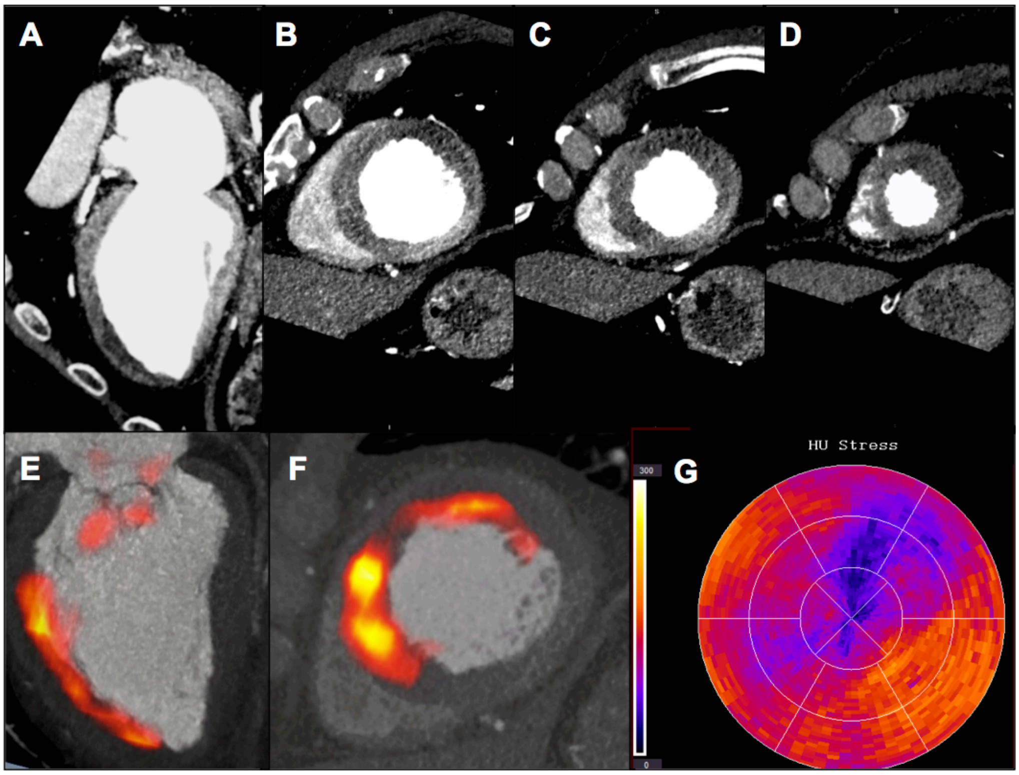

Panels A-D: CT coronary angiogram in long axis (A) and short axis (B-D) reformats. Panels E and F: Fused 18F-fluoride PET CT image in long (E) and short (F) axis reformats. Panel G: 16 segment map of myocardial perfusion during stress. The hypoattenutating, hypoperfused infarcted areas visible on the CT angiogram and perfusion map can be seen to co-localise with intense 18F-fluoride uptake.

Supplementary Tables

Supplementary Table 1: Baseline Scanning Protocol and Radiation Dose Data

	All Patients
Target 18F-FDG Dose (MBq)	200
Actual 18F-FDG Dose (MBq)	199.8 (194.7-203.7)
Actual 18F-FDG Dose range	184-206
Target 18F-Fluoride Dose (MBq)	250
Actual 18F-Fluoride Dose (MBq)	244.5±12.66
Actual 18F-Fluoride Dose range	218 – 266
Target 18F-FDG Uptake Time (min)	90
Actual 18F-FDG Uptake Time	94.1±5.5
Actual 18F-FDG Uptake Time range	84.9-109.8
Target 18F-Fluoride Uptake Time (min)	60
Actual 18F-Fluoride Uptake Time (min)	64.6±5.6
Actual 18F-Fluoride Uptake Time range	56.0 -80.5
Interval between 18F-FDG and 18F-Fluoride (days)	1 (1-2)
**Effective Radiation Dose**	
Average CT dose (mSv)	4.1
Average dose from 18F-Fluoride (mSv)	5.8
Average dose from 18F-FDG (mSv)	4
Average total dose (mSv)	12.9

Parametric data are presented as mean±SD. Non-parametric data are presented as median(IQR). Categorical data are presented as number (percentage). Abbreviations: FDG, fluorodeoxyglucose; MBq, megabecquerels; MSv, millisieverts. *One patient was accidentally given an oncologic dose (368.9MBq) so the actual 18F-FDG dose data are skewed (this patient has been excluded from the range)

Supplementary Table 2: Interobserver And Intraobserver Reproducibility Studies For 18F-Fluoride Uptake At The Internal Carotid Artery And The Blood Pool

Interobserver	Bias	SD of bias	95% LoA	ICC (95%CI)
ICA SUV_{max}	0.04	0.22	-0.39 – 0.47	0.98 (0.96 – 0.99, p<0.001)
ICA $SUV_{meanmax}$	0.04	0.28	-0.50 – 0.59	0.97 (0.93 – 0.99, p<0.001)
ICA SUV_{mean}	-0.002	0.26	-0.51 – 0.51	0.93 (0.84 – 0.97, p<0.001)
Blood pool SUV_{mean}	0.004	0.18	-0.35 – 0.36	0.85 (0.54 – 0.96, p = 0.001)

Intraobserver	Bias	SD of bias	95% LoA	ICC (95%CI)
ICA SUV_{max}	0.04	0.25	-0.44 – 0.53	0.97 (0.93 – 0.99, p<0.001
ICA $SUV_{meanmax}$	0.01	0.22	-0.41 – 0.44	0.97 (0.94 – 0.99, p<0.001
ICA SUV_{mean}	-0.10	0.15	-0.39 – 0.19	0.97 (0.92 – 0.99, p<0.001
Blood pool SUV_{mean}	0.04	0.12	-0.19 – 0.27	0.96 (0.86 – 0.99, p<0.001

ICC – intra-class correlation co-efficient (calculated used a 2-way random effects model to assess absolute agreement)

Supplementary Movie Titles and Legends

Supplementary Movie 1: Explanted Culprit Carotid Plaques As Seen On 18F-Fluoride MicroPET/CT

Two examples of carotid plaques scanned using 18F-Fluoride microPET/CT. The relationship between calcium deposits visible on microCT and the areas of tracer uptake is clearly complex. The tracer is clearly not merely highlighting areas of calcium, but rather micocalcification not resolved by even microCT.

Supplementary Movie 2: Examples Of 18F-Fluoride Uptake Into Areas Of Cerebral Infarction

Three sequential examples of patients with established cerebral infarction showing intense uptake of 18F-Fluoride. Each patient has a run of 2-dimensional axial fused PET/CT images followed by a 3-dimensional render of the ^{18}F-fluoride uptake. The uptake is remarkably high; greater indeed than the adjacent skull.
